# Supplementary material for: Karyotype variation, spontaneous genome rearrangements affecting chemical insensitivity, and expression level polymorphisms in the plant pathogen Phytophthora infestans revealed using its first chromosome-scale assembly
Source: PLoS Pathog. 2022 Oct 10;18(10):e1010869. doi: 10.1371/journal.ppat.1010869 (PMC9584435; doi:10.1371/journal.ppat.1010869)
Supplement: S1 Table — (PDF) [file ppat.1010869.s001.pdf]

S1 Table. Current assemblies of *Phytophthora infestans*

Strain	Technology	Bases in assembly (Mb)	N50 (kb)	Scaffolds	Predicted genes	Reference
1306	PacBio, Illumina, Bionano optical map, Dovetail (proximity ligation), linkage map	247	970	632	19,981	this study
T30-4	Sanger	190[a]	44.5	4,921	17,797	[1]
KR_1_A1	Oxford Nanopore, Illumina	201	291	1,510	20,172	[2]
KR_1_A2	Oxford Nanopore, Illumina	231	152	3,344	23,771	[2]
RC1-10	PacBio	203	120	2,902	n.d.[b]	[3]

[a]Total predicted size of 229 Mb.

[b]Not determined.

References

1. Haas BJ, Kamoun S, Zody MC, Jiang RH, Handsaker RE, Cano LM, et al. Genome sequence and analysis of the Irish potato famine pathogen *Phytophthora infestans*. Nature. 2009;461(7262):393-8.

2. Lee Y, Cho KS, Seo JH, Sohn KH, Prokchorchik M. Improved genome sequence and gene annotation resource for the potato late blight pathogen *Phytophthora infestans*. Mol Plant Microbe In. 2020;33(8):1025-8.

3. Ayala-Usma DA, Cardenas M, Guyot R, De Mares MC, Bernal A, Munoz AR, et al. A whole genome duplication drives the genome evolution of *Phytophthora betacei,* a closely related species to *Phytophthora infestans*. BMC Genomics. 2021;22(1):795.
