# Supplementary material for: Karyotype variation, spontaneous genome rearrangements affecting chemical insensitivity, and expression level polymorphisms in the plant pathogen Phytophthora infestans revealed using its first chromosome-scale assembly
Source: PLoS Pathog. 2022 Oct 10;18(10):e1010869. doi: 10.1371/journal.ppat.1010869 (PMC9584435; doi:10.1371/journal.ppat.1010869)
Supplement: S2 Table — (DOCX) [file ppat.1010869.s002.docx]

**S2 Table. Chromosome and linkage group sizes.**

| Chromosome | |  | Strain 1306 | |  | Strain 618 | |  | Strain 6629 | |  | Strain 550 | |
| --- | --- | --- | --- | --- | --- | --- | --- | --- | --- | --- | --- | --- | --- |
| Name | Size (Mb) |  | Linkage group | Size (cM) |  | Linkage group | Size (cM) |  | Linkage group | Size (cM) |  | Linkage group | Size (cM) |
| chr1 | 22.9 |  | lg1 | 115 |  | lg6 | 138 |  | lg3, 11 | 324 |  | lg2, 6 | 180 |
| chr2 | 21.3 |  | lg2 | 251 |  | lg4 | 104 |  | lg2, 5 | 224 |  | lg4, 7 | 236 |
| chr3 | 16.9 |  | lg3 | 103 |  | lg3 | 67 |  | lg14 | 43 |  | lg10 | 151 |
| chr4 | 16.8 |  | lg4 | 121 |  | lg9 | 78 |  | lg10 | 168 |  | lg9 | 176 |
| chr5 | 14.7 |  | lg5 | 78 |  | lg13 | 67 |  | lg1 | 179 |  | lg3 | 113 |
| chr6 | 14.5 |  | lg6 | 498 |  | lg5 | 103 |  | - | - |  | lg12, 14 | 186 |
| chr7 | 13.8 |  | lg7 | 72 |  | lg1 | 51 |  | lg4 | 75 |  | lg11 | 70 |
| chr8 | 13.5 |  | lg8 | 58 |  | lg10 | 71 |  | lg8 | 49 |  | lg17 | 27 |
| chr9 | 13.4 |  | lg9 | 72 |  | lg12 | 80 |  | lg7 | 92 |  | lg15, 16 | 67 |
| chr10 | 13.2 |  | lg10 | 79 |  | lg2 | 76 |  | lg15, 16 | 128 |  | - | - |
| chr11 | 12.9 |  | lg11 | 72 |  | lg7 | 137 |  | lg13 | 61 |  | lg8 | 119 |
| chr12 | 12.6 |  | lg12 | 57 |  | lg15 | 75 |  | lg12 | 55 |  | - | - |
| chr13 | 11.8 |  | lg13 | 154 |  | lg8 | 76 |  | lg9 | 123 |  | lg1 | 140 |
| chr14 | 10.7 |  | lg14 | 85 |  | lg11 | 111 |  | lg6 | 87 |  | lg11 | 111 |
| chr15 | 10.1 |  | lg15 | 57 |  | lg14 | 56 |  | - | - |  | lg13 | 82 |
| Total | 219.0 |  |  | 1870 |  |  | 1289 |  |  | 1608 |  |  | 1658 |
