# Supplementary material for: Karyotype variation, spontaneous genome rearrangements affecting chemical insensitivity, and expression level polymorphisms in the plant pathogen Phytophthora infestans revealed using its first chromosome-scale assembly
Source: PLoS Pathog. 2022 Oct 10;18(10):e1010869. doi: 10.1371/journal.ppat.1010869 (PMC9584435; doi:10.1371/journal.ppat.1010869)
Supplement: S3 Table — (PDF) [file ppat.1010869.s003.pdf]

S3 Table. Genes differentially expressed between polyploids and diploids

1306 Gene Name	T30-4 Gene Name	Polyploid/diploid (Log 2)	FDR (BH)	Predicted function	Mean RPKM polyploid	Mean RPKM diploid
PI_03chrm0093	PITG_15864	2.23	2E-05	none	33.1	7.06
PI_03chrm0302	PITG_08304	1.54	8E-05	Cyclic nucleotide-dependent kinase	125.4	43.1
PI_03chrm2340	PITG_01526	0.87	1E-04	MAP kinase kinase	36.3	19.9
PI_03chrm2063	PITG_01789	0.79	2E-04	none	26.9	15.6
PI_03chrm2059	PITG_01792	1.33	3E-04	none	24.1	9.6
PI_02chrm0877	PITG_04935	1.2	4E-04	Metallophosphatase	18.9	8.2
PI_02chrm1741	PITG_03669	1.03	4E-04	none	19.1	9.3
PI_02chrm3399	PITG_18446	-0.47	4E-04	26SD proteosome regulatory subunit 6b	117.6	162.9
PI_03chrm1778	PITG_01850	1.06	4E-04	125 kD kinesin	9.5	4.6
PI_09chrm1255	PITG_02501	3.95	4E-04	E3 ubiquitin-protein ligase	375.5	24.3
PI_03chrm0105	PITG_15876	1.11	4E-04	SPRY domain protein	20.6	9.6
PI_04chrm0062	PITG_16427	2.33	4E-04	RxLR effector	53.3	10.6
PI_02chrm1028	PITG_04820	0.67	5E-04	none	76.6	48.1
PI_03chrm0111	PITG_15884	0.88	5E-04	SMC chromosome segregation protein	17.5	9.5
PI_06chrm0053	PITG_16315	0.85	5E-04	none	12.0	6.7
PI_12chrm0975	PITG_06558	-0.6	5E-04	none	14.2	21.5
PI_02chrm1761	PITG_03654	1.33	7E-04	none	6.3	2.5
PI_13chrm0413	PITG_08235	1.18	8E-04	none	89.8	39.6
PI_09chrm1878	PITG_19146	1.94	8E-04	Chromodomain-helicase-DNA binding protein	40.1	10.4
PI_03chrm2034	PITG_01816	1.15	8E-04	Kinase activator MOB1	7.3	3.3
PI_12chrm0560	PITG_07887	-0.67	8E-04	26S proteasome regulatory subunit 6	71.8	114.3
PI_13chrm0414	PITG_08236	1.56	8E-04	Heat shock transcription factor	167.6	56.8
PI_077Fc020	PITG_17400	1.73	8E-04	HSP40-class protein chaperone	225.7	68.0
PI_03chrm2037	PITG_01814	0.71	9E-04	ELMO domain GTPase-activating protein	121.5	74.2
PI_0131c021	PITG_15818	0.92	1E-03	Acyl-CoA-binding protein	47.6	25.1
PI_02chrm0876	PITG_04936	0.79	1E-03	Zinc finger protein	14.8	8.6
PI_05chrm0874	PITG_09346	1.47	1E-03	none	33.1	12.0
PI_06chrm0233	PITG_00208	0.64	1E-03	Never in mitosis A-related kinase	145.4	93.3
PI_08chrm0136	PITG_13379	1.3	1E-03	RhoGEF domain protein	59.5	24.2
PI_06chrm2476	PITG_12619	1.23	1E-03	Crinkler family protein	20.9	8.9
PI_03chrm1657	PITG_01957	0.73	1E-03	Serine/threonine protein phosphatase	27.2	16.4
PI_03chrm0110	PITG_15883	0.91	1E-03	Myotubularin-like phosphatase	11.6	6.2

PI_03chrm1708	PITG_01920	0.58	1E-03	Ubiquitin carboxyl-terminal hydrolase	21.8	14.6
PI_01chrm4439	PITG_12235	0.69	2E-03	E3 ubiquitin-protein ligase	40.9	25.3
PI_08chrm0320	PITG_13286	1.8	2E-03	none	80.7	23.2
PI_04chrm0062	PITG_16418	2.53	2E-03	RxLR protein	35.6	6.2
PI_03chrm2276	PITG_01589	0.66	2E-03	none	21.6	13.7
PI_06chrm0390	PITG_00190	0.62	2E-03	FAD-binding protein	20.6	13.4
PI_09chrm0313	PITG_10763	1.65	2E-03	Lysophospholipid acyltransferase	78.3	25.0
PI_09chrm0860	PITG_02842	0.96	2E-03	CDP-alcohol phosphatidyltransferase	40.4	20.8
PI_07chrm0247	PITG_05729	1.75	2E-03	none	71.6	21.3
PI_05chrm1297	PITG_11583	2.86	2E-03	Ring finger protein	108.6	15.0
PI_09chrm0043	PITG_18173	0.95	2E-03	none	5.3	2.7
PI_05chrm1298	PITG_11584	2.86	2E-03	BAG domain proliferation and stress protein	229.8	31.6
PI_08chrm0258	PITG_21770	1.55	2E-03	none	33.7	11.5
PI_03chrm1258	PITG_10154	1.94	2E-03	MYND domain zinc finger protein	104.6	27.3
PI_03chrm2253	PITG_01614	0.67	2E-03	Sodium/hydrogen exchanger	39.6	24.9
PI_03chrm0200	PITG_15961	0.93	2E-03	none	32.2	16.9
PI_04chrm0062	PITG_16245	2.55	2E-03	none	35.8	6.1
PI_02chrm3931	PITG_00845	1.57	2E-03	Cyclic nucleotide phosphodiesterase	25.2	8.5
PI_02chrm1641	PITG_03811	1.32	3E-03	none	118.2	47.4
PI_09chrm0230	PITG_10686	1.06	3E-03	none	4.0	1.9
PI_03chrm0712	PITG_21505	2.49	3E-03	none	21.1	3.8
PI_04chrm0744	PITG_10271	0.92	3E-03	none	10.6	5.6
PI_03chrm2274	PITG_01591	0.73	3E-03	Nucleopore protein	72.4	43.6
PI_12chrm0715	PITG_18648	-0.72	3E-03	Proteasome regulator ADRM1	15.5	25.5
PI_03chrm2028	PITG_01821	0.74	3E-03	Rab-GAP TBC domain-containing protein	29.4	17.6
PI_12chrm0092	PITG_08136	-0.57	3E-03	Bromodomain acetyl lysine binding protein	23.3	34.6
PI_03chrm1370	PITG_02050	2.22	3E-03	Glycosyl transferase	34.8	7.5
PI_12chrm1417	PITG_06171	-0.39	3E-03	U2 snRNP spliceosome subunit	35.9	47.1
PI_11chrm0409	PITG_14550	1.27	3E-03	Tudor domain RNA-binding protein	11.1	4.6
PI_03chrm1037	PITG_09997	0.55	3E-03	Cytidyly phospholtransferase	23.0	15.7
PI_03chrm2040	PITG_01811	0.83	3E-03	none	8.8	4.9
PI_09chrm0319	PITG_10768	1.66	3E-03	DNA-binding zinc finger protein	25.6	8.1
PI_03chrm2190	PITG_01674	0.45	3E-03	P-loop protein	36.0	26.4
PI_06chrm1749	PITG_05233	0.74	3E-03	CLIP-associating protein	35.8	21.5
PI_03chrm1288	PITG_10164	0.55	3E-03	Sir1 histone deacetylase	20.3	13.9
PI_03chrm1259	PITG_10153	2.3	3E-03	none	169.1	34.3

PI_01chrm1408	PITG_11029	0.64	3E-03	DNAJ-class protein chaperone	53.8	34.5
PI_02chrm2850	PITG_18045	0.59	3E-03	MAP kinase-activated protein kinase	102.5	68.1
PI_02chrm3904	PITG_00867	0.58	3E-03	LA motif RNA binding protein	47.5	31.8
PI_03chrm1045	PITG_10002	1.17	3E-03	Leucine zipper transcription factor	51.3	22.8
PI_06chrm1269	PITG_05328	0.98	3E-03	Ankyrin-like protein	66.8	33.9
PI_12chrm0671	PITG_22839	-0.61	3E-03	SMC chromosome segregation protein	23.9	36.5
PI_03chrm0310	PITG_08311	1.28	3E-03	none	1276.7	525.7
PI_11chrm0343	PITG_21331	0.6	3E-03	none	23.2	15.3
PI_03chrm0312	PITG_08312	1.26	3E-03	Tyr protein kinase	116.2	48.5
PI_0366c004	PITG_22487	0.65	3E-03	RNA binding factor subunit	9.0	5.8
PI_075Fc004	PITG_03695	1.43	4E-03	none	1062.9	394.5
PI_09chrm0315	PITG_10764	1.51	4E-03	none	17.4	6.1
PI_12chrm0540	PITG_07903	-0.59	4E-03	26S proteasome regulatory subunit 8	69.9	105.2
PI_04chrm1272	PITG_17391	0.9	4E-03	Heat shock transcription factor	7.0	3.7
PI_0137c017	PITG_19674	0.67	4E-03	none	12.0	7.6
PI_03chrm0118	PITG_15891	0.88	4E-03	Palmitoyltransferase	57.4	31.2
PI_06chrm1196	PITG_00792	1.61	4E-03	none	143.7	47.1
PI_02chrm1148	PITG_04701	0.9	4E-03	Heat shock transcription factor	38.9	20.8
PI_02chrm1506	PITG_03943	2.04	4E-03	Methtyltransferase domain protein	48.0	11.7
PI_02chrm2642	PITG_03104	0.6	4E-03	Choline transporter	77.1	50.8
PI_0387c007	PITG_06759	0.73	4E-03	none	40.8	24.6
PI_04chrm0834	PITG_10375	4.16	4E-03	none	84.3	4.7
PI_06chrm1105	PITG_00718	0.77	4E-03	Sir2 histone deacetylase	12.9	7.5
PI_15chrm0204	PITG_15670	2.02	4E-03	Rab5 effector protein	16.4	4.1
PI_02chrm3146	PITG_01079	1.14	4E-03	Rab 5 effector protein	5.7	2.6
PI_03chrm0138	PITG_15906	0.84	4E-03	DNA repair protein RAD51	58.9	32.9
PI_03chrm1667	PITG_01949	0.58	4E-03	Glycosyl hydrolase	8.4	5.6
PI_03chrm0072	PITG_15846	1.29	5E-03	none	159.5	65.2
PI_03chrm1343	PITG_02027	0.63	5E-03	E3 ubiquitin-protein ligase	31.3	20.2
PI_03chrm2091	PITG_01761	1.25	5E-03	Proline dehydrogenase	89.3	37.5
PI_03chrm1183	PITG_10117	0.68	5E-03	ABC transporter	41.7	26.0
PI_03chrm1035	PITG_09995	2.07	5E-03	none	91.6	21.8
PI_12chrm0608	PITG_07846	-0.61	5E-03	Glycylpeptide N-tetradecanoyltransferase	65.2	99.5
PI_02chrm1147	PITG_04702	0.88	5E-03	Caspase 8-associated protein	65.9	35.8
PI_07chrm0522	PITG_05929	-0.95	5E-03	GOLD domain transmembrane protein	10.5	20.4
PI_12chrm0289	PITG_07995	-0.6	5E-03	Cyclin-dependent kinase	18.3	27.8

PI_06chrm1218	PITG_00809	0.7	5E-03	none	8.1	5.0
PI_13chrm0204	PITG_16916	1.8	5E-03	Serine/threonine protein kinase	78.3	22.5
PI_02chrm1788	PITG_03523	0.4	5E-03	Transcription factor IIH subunit 1	18.6	14.1
PI_03chrm1650	PITG_01930	0.87	5E-03	cAMP kinase regulatory subunit	34.2	18.7
PI_11chrm0343	PITG_14605	0.56	5E-03	none	36.3	24.6
PI_02chrm3954	PITG_17238	0.53	5E-03	none	11.1	7.7
PI_09chrm1662	PITG_02204	1.28	5E-03	Cyclic nucleotide phosphodiesterase	11.9	4.9
PI_09chrm0861	PITG_02841	1.22	5E-03	Cysteine protease	54.3	23.3
PI_02chrm2437	PITG_00971	0.75	6E-03	none	16.9	10.0
PI_03chrm0786	PITG_08613	1.1	6E-03	Endoglucanase	182.0	84.9
PI_03chrm2066	PITG_01785	0.63	6E-03	none	30.2	19.5
PI_03chrm2623	PITG_16069	2.3	6E-03	HSP100-class protein chaperone	138.7	28.2
PI_04chrm0717	PITG_10239	0.92	6E-03	Alpha-type protein kinase	24.1	12.7
PI_06chrm1549	PITG_05286	0.45	6E-03	none	29.6	21.7
PI_11chrm0368	PITG_14581	1.43	6E-03	none	33.4	12.4
PI_12chrm0090	PITG_08138	-0.72	6E-03	none	37.7	62.1
PI_14chrm0346	PITG_13056	-0.97	6E-03	Mitochondrial carrier protein	24.9	48.9
PI_04chrm0834	PITG_10370	4.19	6E-03	none	66.0	3.6
PI_11chrm0886	PITG_09723	0.9	6E-03	Thioredoxin	57.7	30.9
PI_02chrm3085	PITG_01024	0.83	6E-03	none	15.4	8.7
PI_01chrm1889	PITG_00015	1.03	6E-03	HTH DNA-binding protein	25.4	12.5
PI_03chrm1700	PITG_01923	0.35	6E-03	PWWP histone methyl-lysine binding protein	70.0	54.9
PI_09chrm0235	PITG_10690	0.42	6E-03	none	10.7	8.0
PI_12chrm1378	PITG_06202	2.02	6E-03	none	360.2	88.8
PI_02chrm2438	PITG_00972	1.32	6E-03	Aldehyde reductase	35.0	14.0
PI_03chrm2341	PITG_01525	0.66	6E-03	none	120.8	76.5
PI_06chrm1556	PITG_05291	0.99	6E-03	Inward rectifier potassium channel	22.0	11.1
PI_03chrm0119	PITG_19905	0.92	6E-03	Serine/threonine protein kinase	61.0	32.2
PI_12chrm1379	PITG_06201	1.93	6E-03	TATA-box-binding protein TFIID	141.2	37.1
PI_01chrm4443	PITG_12231	2.11	6E-03	Diacylglycerol phosphatase	99.7	23.1
PI_06chrm1544	PITG_05278	0.62	6E-03	PDH zinc finger protein	10.1	6.6
PI_03chrm2137	PITG_01719	0.42	6E-03	Vaculolar sorting protein Vps39	48.7	36.4
PI_01chrm1843	PITG_11264	0.8	6E-03	none	42.8	24.6
PI_03chrm0119	PITG_15892	0.98	6E-03	Serine/threonine protein kinase	102.7	52.1
PI_15chrm0562	PITG_15238	1.77	6E-03	none	18.4	5.4
PI_02chrm2320	PITG_03592	1.46	6E-03	protein phosphatase regulatory subunit	88.6	32.2

PI_02chrm2319	PITG_03593	1.55	6E-03	Alanine aminotransferase	235.2	80.3
PI_12chrm0965	PITG_22394	-0.62	6E-03	EH domain protein	4.0	6.1
PI_01chrm4373	PITG_12296	1.41	7E-03	none	6.8	2.6
PI_03chrm1273	PITG_10141	0.65	7E-03	Adenylyl/guanylyl cyclase	33.2	21.1
PI_03chrm2027	PITG_01822	0.6	7E-03	none	59.2	39.1
PI_03chrm2079	PITG_01774	0.65	7E-03	none	13.6	8.7
PI_03chrm2215	PITG_01650	0.65	7E-03	E3 ubiquitin-protein ligase	10.6	6.8
PI_03chrm2250	PITG_01617	0.58	7E-03	none	43.6	29.2
PI_03chrm2270	PITG_01599	0.49	7E-03	Coronin actin family cytoskeletal protein	166.3	118.4
PI_06chrm1335	PITG_18959	0.98	7E-03	Serine/threonine protein kinase	29.2	14.8
PI_06chrm1546	PITG_05282	0.69	7E-03	Phosphatidylinositol 4-phosphate 5-kinase	56.6	35.1
PI_08chrm0852	PITG_01317	0.59	7E-03	ABC-type protein kinase	23.3	15.5
PI_08chrm0909	PITG_01280	-0.42	7E-03	TOM1 family ubiquitin-binding protein	33.7	45.1
PI_08chrm0983	PITG_01208	-0.78	7E-03	Thiolase	147.9	254.0
PI_10chrm1015	PITG_05168	0.99	7E-03	none	10.9	5.5
PI_13chrm0474	PITG_15149	0.64	7E-03	YSC84-like actin polymerization protein	55.8	35.8
PI_13chrm0539	PITG_15085	-0.68	7E-03	none	4.1	6.6
PI_14chrm0404	PITG_13014	-1.18	7E-03	rRNA binding protein	83.6	189.5
PI_04chrm1016	PITG_10488	2.01	7E-03	Serine/threonine protein kinase	13.6	3.4
PI_0697c002	PITG_16618	0.73	7E-03	RNA binding factor subunit	13.2	8.0
PI_04chrm0784	PITG_10308	0.72	7E-03	none	40.9	24.8
PI_03chrm0158	PITG_15922	1.11	7E-03	SMC chromosome segregation protein	20.9	9.7
PI_12chrm0810	PITG_06656	-0.64	7E-03	ATP-binding protein	4.1	6.4
PI_03chrm0198	PITG_15959	0.72	7E-03	none	43.1	26.2
PI_14chrm0016	PITG_13178	-1.07	7E-03	Fatty acid elongase	73.4	154.1
PI_11chrm0366	PITG_14582	1.04	7E-03	Phosphodiesterase	12.0	5.8
PI_03chrm2272	PITG_01598	0.42	7E-03	Mannose-binding protein	67.0	50.1
PI_03chrm2427	PITG_09544	1.48	7E-03	none	18.5	6.6
PI_03chrm2284	PITG_01580	0.43	7E-03	Signal peptidase 25 kDa subunit	89.7	66.6
PI_02chrm0579	PITG_11792	0.86	8E-03	none	160.4	88.4
PI_04chrm0280	PITG_16495	1.54	8E-03	ABC transporter	11.9	4.1
PI_05chrm1107	PITG_14214	2.4	8E-03	none	30.4	5.8
PI_06chrm0438	PITG_00147	1.35	8E-03	none	39.2	15.4
PI_06chrm0414	PITG_00167	1.3	8E-03	Cyclic nucleotide phosphodiesterase	47.3	19.2
PI_12chrm1307	PITG_06272	-0.66	8E-03	ADP ribosylation factor GTPase-activating protein	10.9	17.2

PI_03chrm0664	PITG_08489	0.48	8E-03	none	30.0	21.5
PI_06chrm1570	PITG_05303	0.59	8E-03	none	301.7	200.4
PI_02chrm3798	PITG_00964	0.91	8E-03	bZip transcription factor	76.2	40.6
PI_03chrm2201	PITG_01664	1.38	8E-03	none	33.0	12.7
PI_03chrm2628	PITG_16074	2.03	8E-03	HSP100-class protein chaperone	106.5	26.1
PI_07chrm1360	PITG_12578	-1.09	8E-03	Acyl-CoA desaturase	91.4	194.5
PI_12chrm0747	PITG_06695	-0.55	8E-03	Serine/arginine-rich splicing factor 4	35.7	52.3
PI_02chrm3984	PITG_17265	0.72	9E-03	none	9.9	6.0
PI_02chrm3778	PITG_00989	0.67	9E-03	Jumonji family transcription factor	22.7	14.3
PI_03chrm2214	PITG_01651	0.87	9E-03	RAB-GTPase	36.0	19.7
PI_08chrm0010	PITG_13441	-0.32	9E-03	Phosphatidylinositol 4-kinase	7.7	9.6
PI_03chrm2283	PITG_01581	0.37	9E-03	Mitotic spindle assembly checkpoint protein MAD2A	52.7	40.7
PI_0697c002	PITG_12094	0.36	9E-03	Serine/threonine protein kinase	23.7	18.4
PI_06chrm0082	PITG_16338	1.09	9E-03	none	61.1	28.7
PI_14chrm0281	PITG_20412	-1.4	9E-03	Putative GPI-anchored elicitin protein	28.2	74.5
PI_03chrm2427	PITG_20979	1.47	9E-03	none	13.0	4.7
PI_03chrm2364	PITG_01508	0.46	9E-03	GYF domain protein	55.7	40.5
PI_09chrm0941	PITG_02776	1.01	9E-03	Inositol phosphate phosphatase	3.8	1.9
PI_14chrm1009	PITG_17049	1.05	9E-03	Multidrug resistance protein	38.7	18.7
PI_04chrm0788	PITG_10310	1.62	9E-03	Histone 2A deubiquitinase	38.4	12.5
PI_04chrm1013	PITG_10486	1.39	9E-03	DNA replication GINS complex protein	9.2	3.5
PI_12chrm0727	PITG_18641	-0.41	9E-03	none	19.0	25.2
PI_02chrm0781	PITG_17894	2.92	9E-03	Zinc finger protein	62.3	8.2
PI_03chrm1090	PITG_10040	0.96	9E-03	none	10.2	5.2
PI_03chrm2359	PITG_01512	1.47	9E-03	none	12.2	4.4
PI_075Fc031	PITG_03753	0.86	9E-03	E3 ubiquitin-protein ligase	36.8	20.3
PI_03chrm2065	PITG_01786	0.31	9E-03	none	21.1	17.0
PI_05chrm0600	PITG_09194	2.6	9E-03	Aquaporin	285.3	47.1
PI_09chrm0264	PITG_10722	1.5	9E-03	none	29.8	10.5
PI_11chrm0746	PITG_18001	0.83	9E-03	Polyubiquitin	1107.4	622.9
PI_03chrm2104	PITG_01749	0.36	9E-03	50S ribosomal protein L9	22.2	17.3
PI_08chrm0977	PITG_01217	-1.03	1E-02	50S ribosomal protein L9	44.8	91.5
PI_09chrm0774	PITG_02921	-0.48	1E-02	tRNA synthetase	69.6	97.1
PI_09chrm0775	PITG_02922	-0.53	1E-02	Polycystin cation channel protein	35.1	50.7
PI_03chrm1375	PITG_02055	0.68	1E-02	TPR repeat protein	50.3	31.4

PI_06chrm0227	PITG_00214	0.34	1E-02	none	21.9	17.3
PI_01chrm3551	PITG_06862	1.98	1E-02	ABC transporter	99.8	25.3
PI_02chrm2116	PITG_03265	0.49	1E-02	Sodium/hydrogen exchanger	15.6	11.1
PI_09chrm1180	PITG_02566	0.56	1E-02	Condensin	10.5	7.1
PI_02chrm1947	PITG_03416	0.57	1E-02	Cysteine protease	63.4	42.7
