# Supplementary material for: Karyotype variation, spontaneous genome rearrangements affecting chemical insensitivity, and expression level polymorphisms in the plant pathogen Phytophthora infestans revealed using its first chromosome-scale assembly
Source: PLoS Pathog. 2022 Oct 10;18(10):e1010869. doi: 10.1371/journal.ppat.1010869 (PMC9584435; doi:10.1371/journal.ppat.1010869)
Supplement: S6 Table — (DOCX) [file ppat.1010869.s006.docx]

**S6 Table. Primer sets**

| Name | Sequence (5' to 3') | Application |
| --- | --- | --- |
| 15955 F,R | ATGCTCGTCTCGATCGTCTT, GATGCGCTGACGCACTATTA | RT-qPCR |
| 13763 F,R | GGGTAAGTCGTTCGCTGAAG, GGGTAAGTCGTTCGCTGAAG | RT-qPCR |
| 05712 F,R | GTCCGAGCTCTCCAACACTC, ACGCCTTGTGTGGTATGGAT | RT-qPCR |
| 13006 F,R | GTCATGACGGCCGATATCTT, CGATACTCCCACGATGAGGT | RT-qPCR |
| 07424 F,R | GTGCCGGATATGCAAGAAGT, GAACGTACCATCCGGAAAGA | RT-qPCR |
| 14253 F,R | ACGGTGGGTTGTCAATGTTT, GAAATCATACCCGCGAGAAA | RT-qPCR |
| 16698 F,R | CTGTGTCACGAACCCATACG, CGCTCTTGTTCTTGCTCCTC | RT-qPCR |
| 09862 F,R | ATTCGTGGGAGCACCTTGTA, ACTTCCCTCCACTCGTCAAG | RT-qPCR |
| CHR5 F,R | GTACGGGTGGAATTTGAGGC, TTGTTTGTATTGCGCGTCCA | qPCR |
| CHR9 F,R | CTCCTTCGACGCCAATTCTG, CAGATGAATGGCTTGCGTGT | qPCR |
| CHR10 F, R | TGGTGTTCAGTGCCATTGTG, GCGACGTTGATCAGACACAG | qPCR |
| LF1_10087 F, R | ACGCCAAGTCAAGCAAAGA, CTCTTCGTCTTCGGGCTTTC | HRM Chr3 |
| LF2_01520 | ATCGCATCGTGGGTAGTATTG, CGAACTCCCATTCTCCCTTAAA | HRM Chr3 |
| CNV_16115 | AGCTGACTTCAGATGAGCAAG, TGTGGGTTCTACTGGGTTAGA | HRM Chr3 |
