# Supplementary figures and images for: Karyotype variation, spontaneous genome rearrangements affecting chemical insensitivity, and expression level polymorphisms in the plant pathogen Phytophthora infestans revealed using its first chromosome-scale assembly
Source: PLoS Pathog. 2022 Oct 10;18(10):e1010869. doi: 10.1371/journal.ppat.1010869 (PMC9584435; doi:10.1371/journal.ppat.1010869)

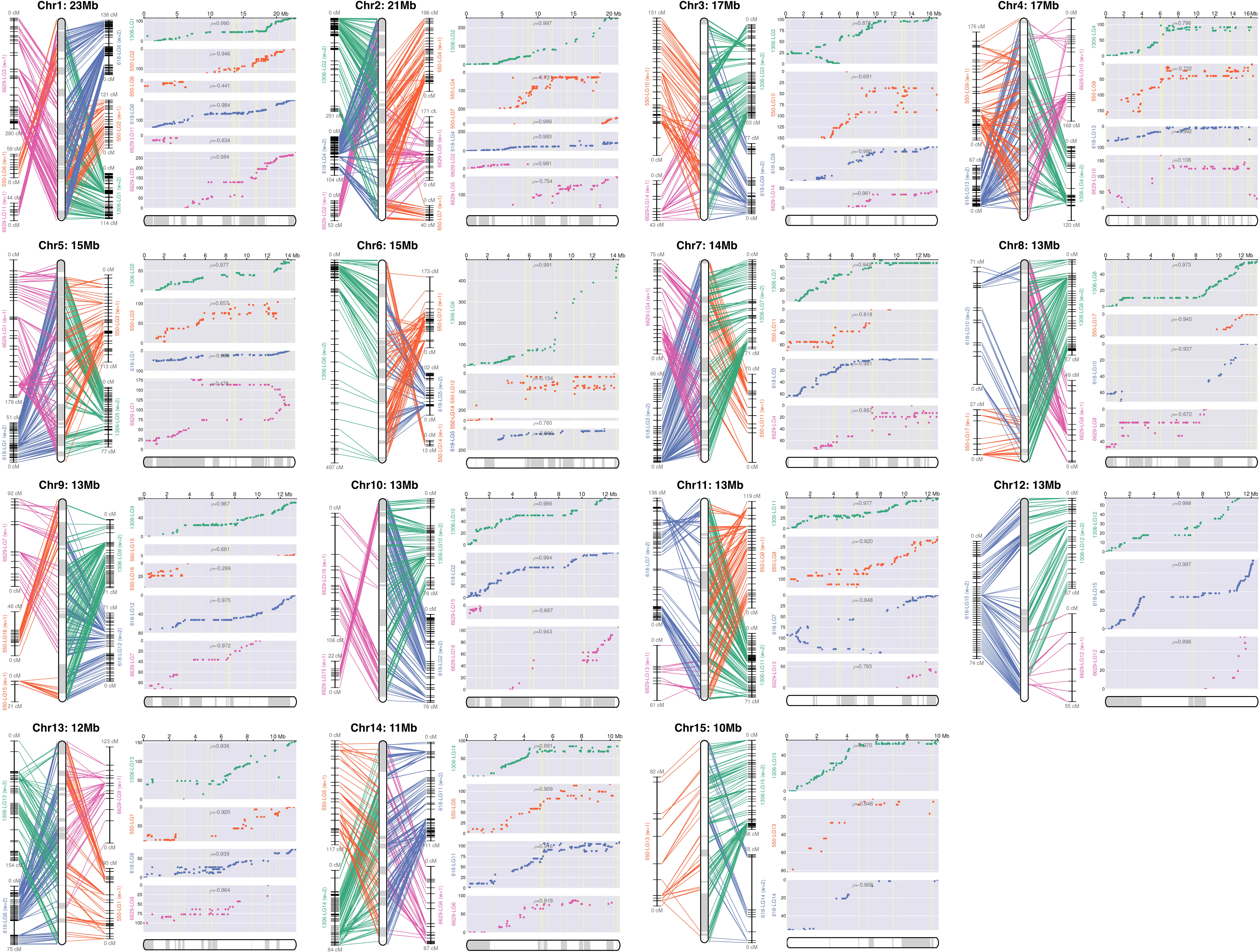

Supplement: S1 Fig — Indicated are genetic maps of each parent (left) and plots of genetic versus physical distance in each parent (right), as described in the legend to Fig 3. (TIF) [file ppat.1010869.s007.tif]

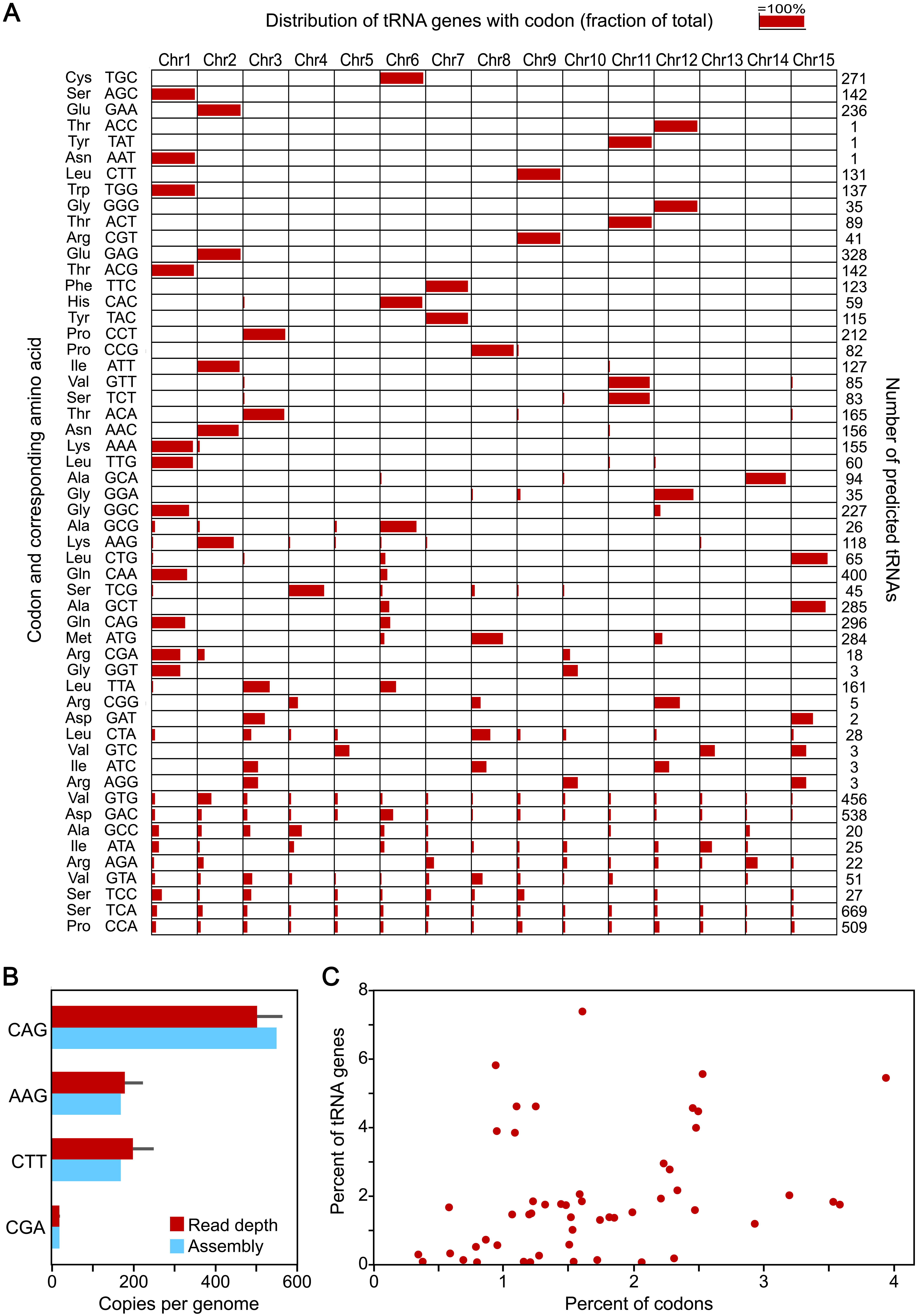

Supplement: S2 Fig — A, genomic distribution of tRNA genes, with red bars representing the fraction of tRNAs corresponding to each codon per chromosome. The total number of genes per family on the chromosomes is shown on the right. The analysis excluded 1,292 genes predicted to encode pseudogenes. B, copy numbers of representative families predicted from the assembly (blue) and calculated by read depth analysis (red). Codons bound by the tRNAs are indicated. C, relationship between gene copy number and the frequency of the corresponding codons in genes. (TIF) [file ppat.1010869.s008.tif]

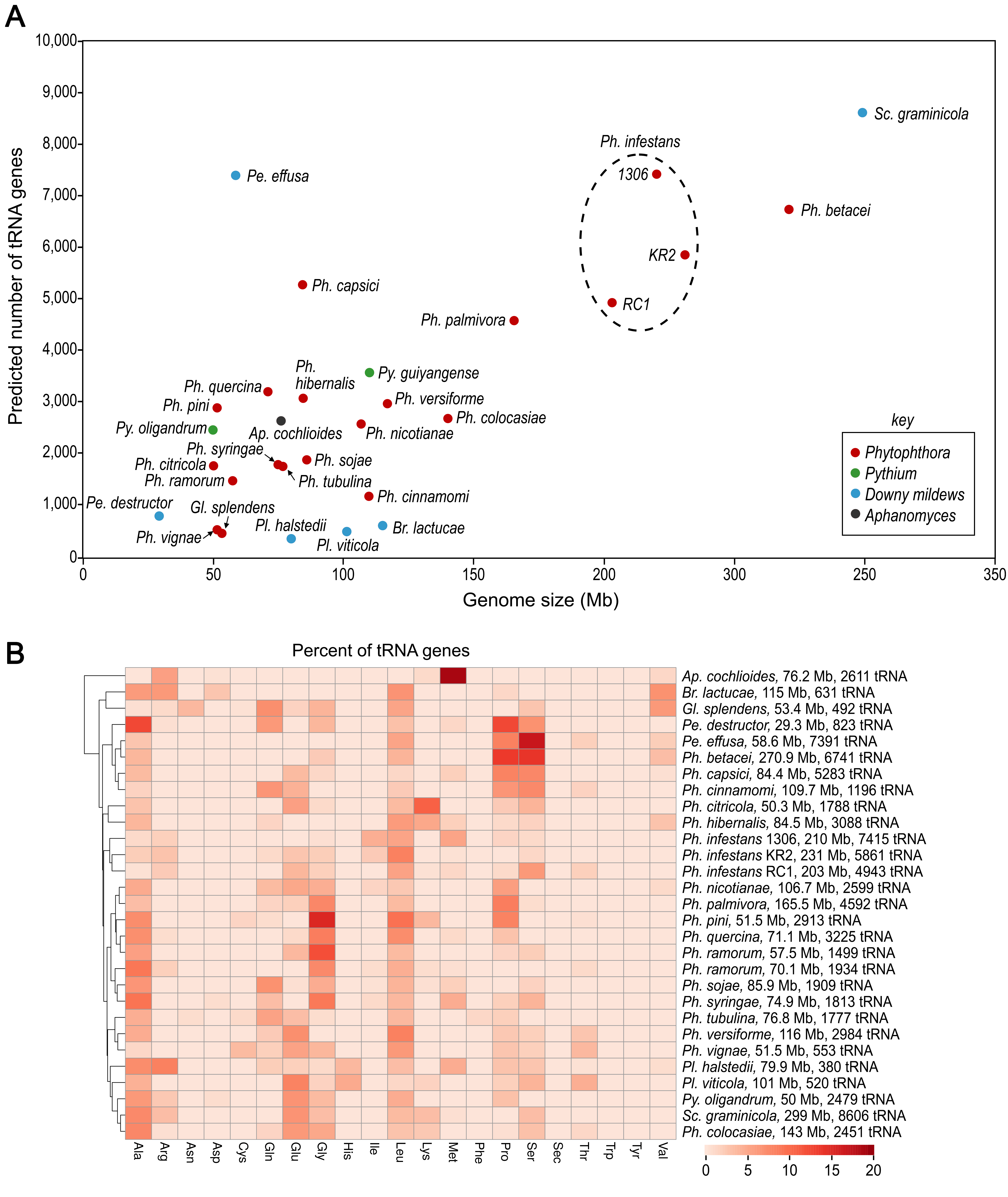

Supplement: S3 Fig — A, number of tRNA genes predicted per species plotted as a function of genome size. The species belong to the genus Phytophthora (Ph. prefix, red-filled circles), the Pythium-Globisporangium group (Py. and Gl., green), the downy mildew group including Bremia, Peronospora, Plasmopara, and Sclerospora (Br., Pe., Pl., Sc., cyan), and Aphanomyces (Ap., black). B, relative frequency of tRNA genes corresponding to the 20 amino acids plus selenocysteine (Sec) in each genome. This analysis was limited to the largest assembly based on long reads available in NCBI Genome, except for P. infestans which includes three assemblies. Accession numbers of the assemblies are GCA_001482985.1, Ph. nicotianae; GCA_001887855.2, Sc. graminicola; GCA_001974925.1, Pl. viticola; GCA_003730235.1, Py. guiyangense; GCA_003956735.1, Ph. ramorum; GCA_004359215.2, Br. lactucae; GCA_004380875.1, Pl. halstedii; GCA_005966545.1, Py. oligandrum; GCA_006386115.1, Gl. (formerly Py.) splendens; GCA_007655245.1, Ph. citricola; GCA_009848525.1, Ph. sojae; GCA_011316315.1. Ph. infestans RC1-10; GCA_011320135.1, Ph. betacei; GCA_011800735.1, Pe. destructor; GCA_012552325.1, Ph. infestans KR2; GCA_012656075.1, Ph. hibernalis; GCA_012656105.1, Ph. syringae; GCA_014706105.1, Ph. quercina; GCA_014706135.1, Ph. tubulina; GCA_014706215.1, Ph. versiforme; GCA_016169925.1, Ph. vignae; GCA_016618375.1, Ph. capsici; GCA_018691715.1, Ph. cinnamomi; GCA_019828595.1, Ap. cochlioides; GCA_020226015.1, Ph. colocasiae; GCA_021491655.1, Pe. effusa; and GCA_023611945.1, Ph. pini. (TIF) [file ppat.1010869.s009.tif]

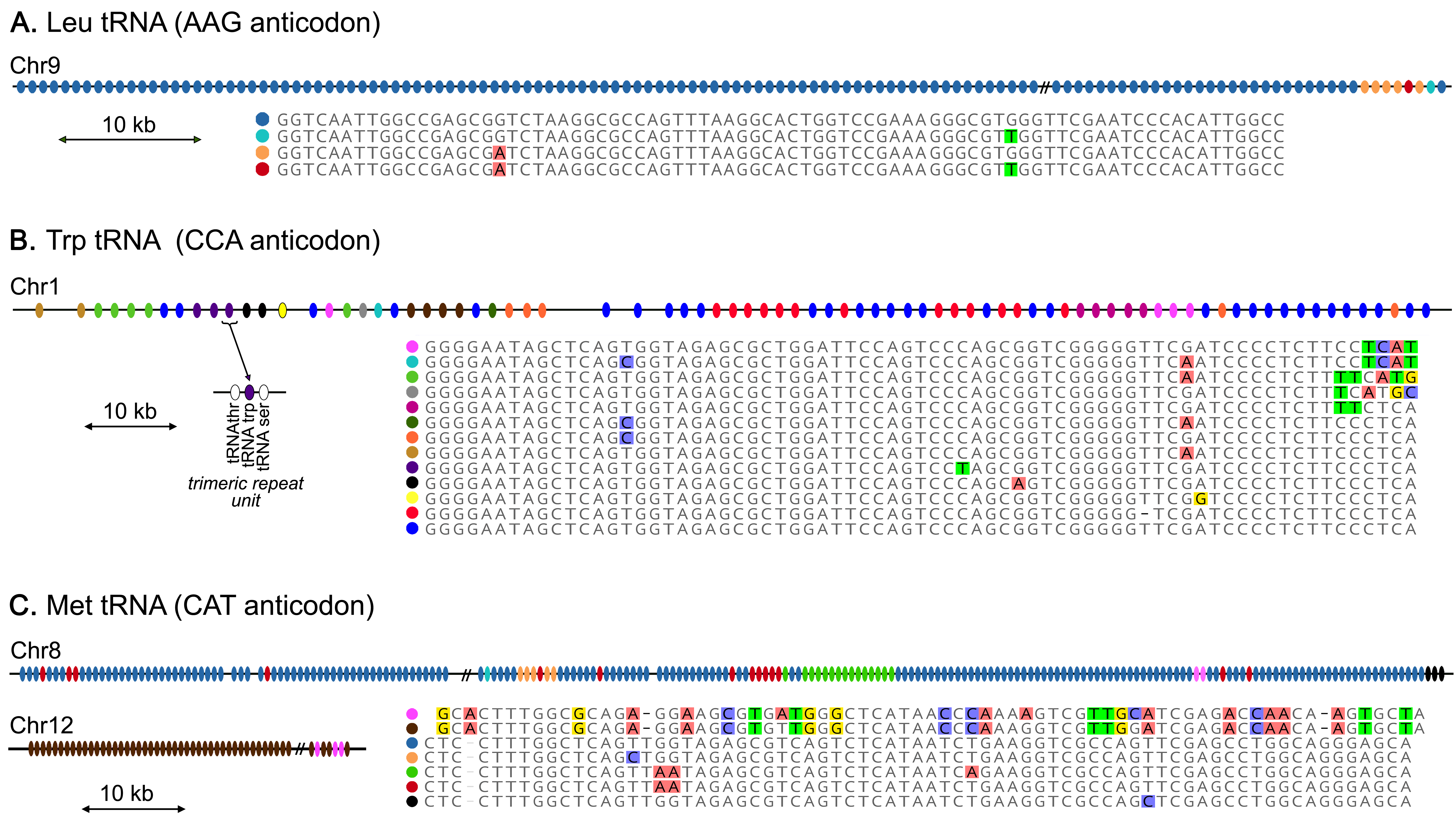

Supplement: S4 Fig — A, Sequence variation within array of genes encoding tRNAleu (AAG anticodon) on Chr9. The genotype of each copy (ellipses) is color-coded according to the sequences in the alignment, in which bases diverging from the genome-wide consensus are colored. The hashmark on the chromosome represents a gap in the assembly. B, same as panel A but showing genes encoding tRNAmet (CAT anticodon) on Chr8 and Chr12. C, same as panel A but for a representative portion of the array encoding tRNAtrp (CCA anticodon) on Chr1. As illustrated below the chromosome, this tRNA occurs as part of a trimeric repeat encoding tRNAser, tRNAtrp, tRNAthr. Variation within the ser- and thr tRNAs is not shown. (TIF) [file ppat.1010869.s010.tif]

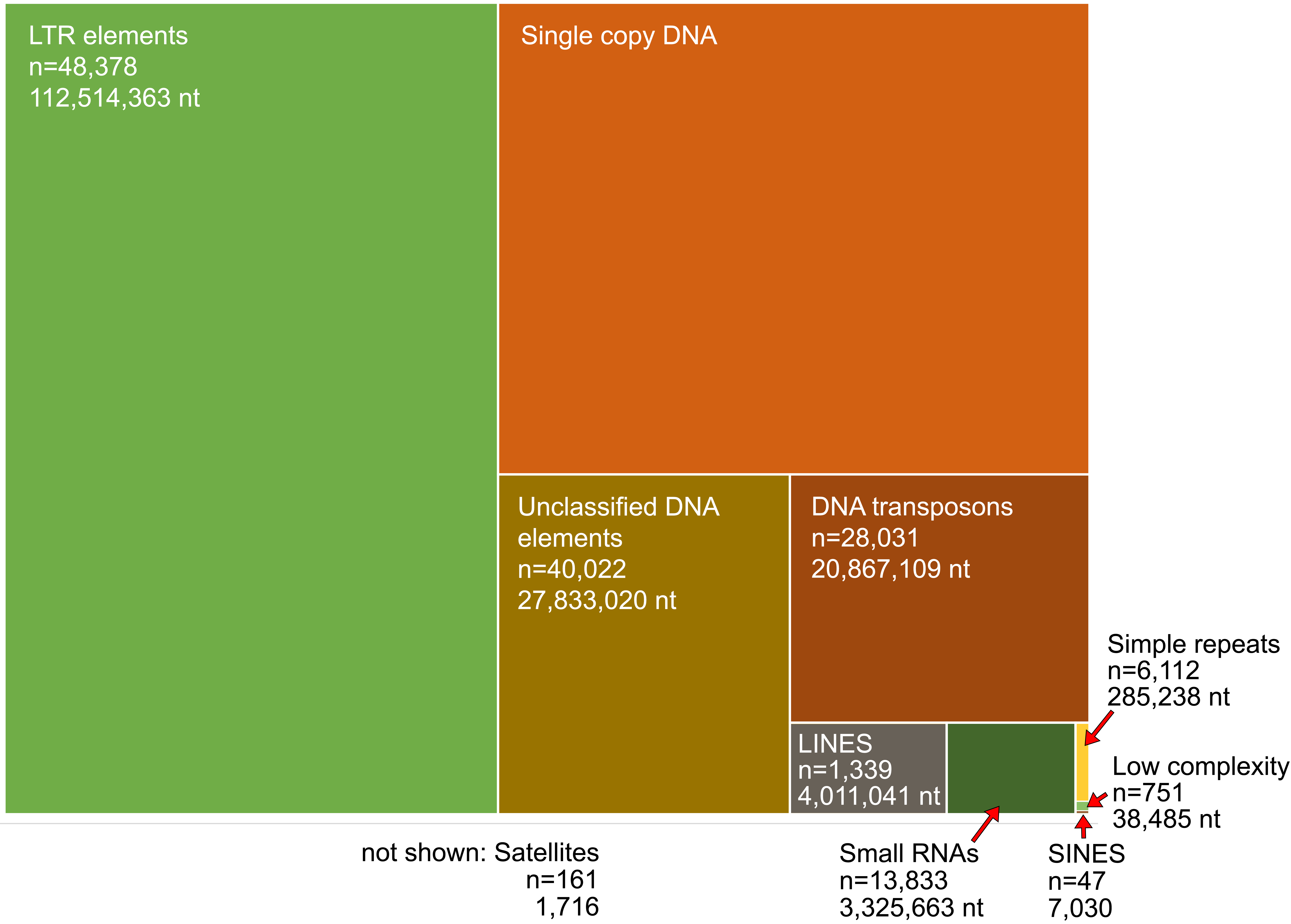

Supplement: S5 Fig — Indicated are the number and total size in nt of LTR retrotransposons, DNA transposons, LINES, SINESs, simple repeats and satellites, genes encoding small RNAs (including tRNA, rRNA, snRNA, scRNA, srpRNA), and elements not classified by RepeatMasker. (TIF) [file ppat.1010869.s011.tif]

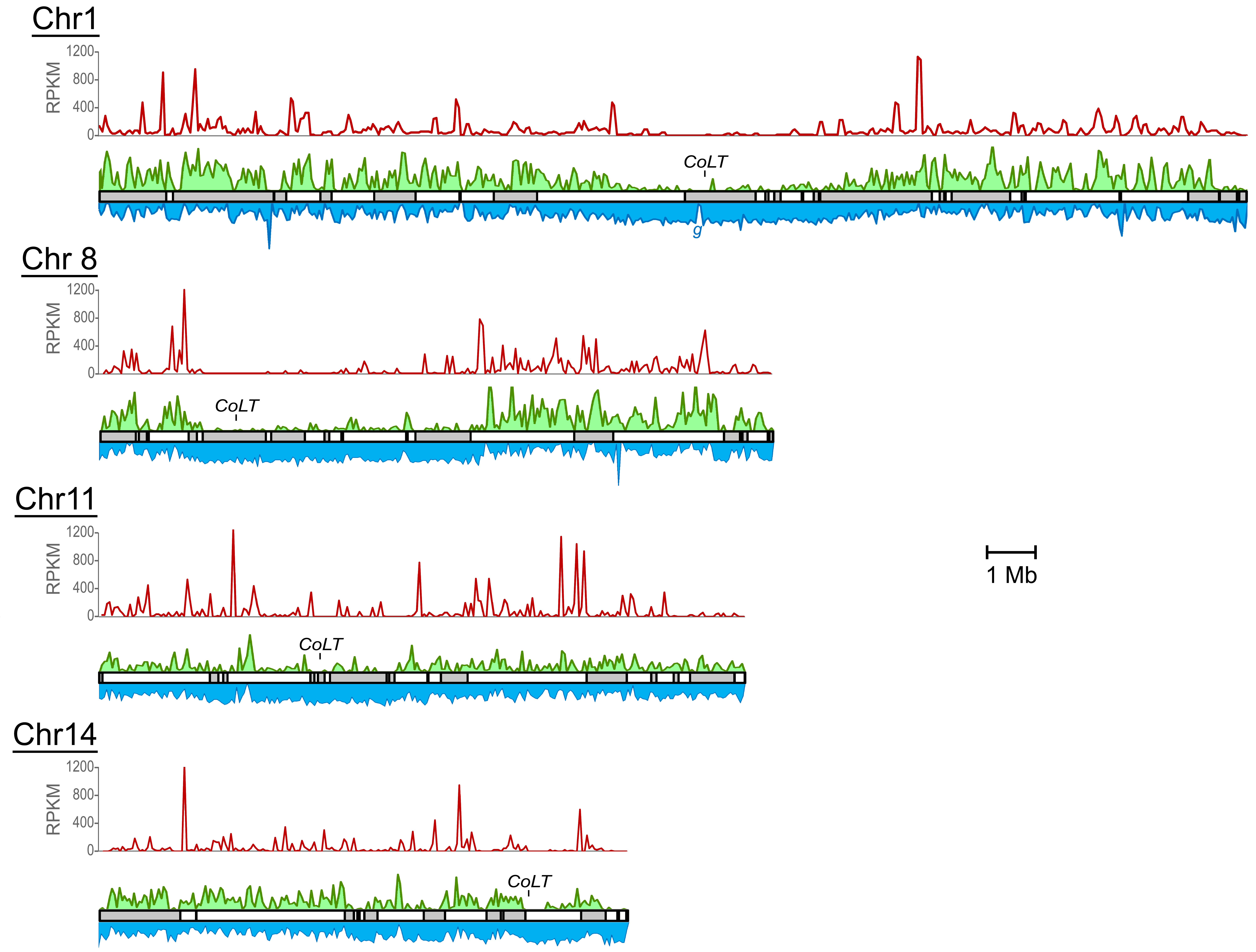

Supplement: S6 Fig — Shown for representative chromosomes are density graphs of mRNA (red), displayed as RPKM for each 50-kb bin. The green and blue graphs are densities of genes and repetitive DNA taken from Fig 2. (TIF) [file ppat.1010869.s012.tif]

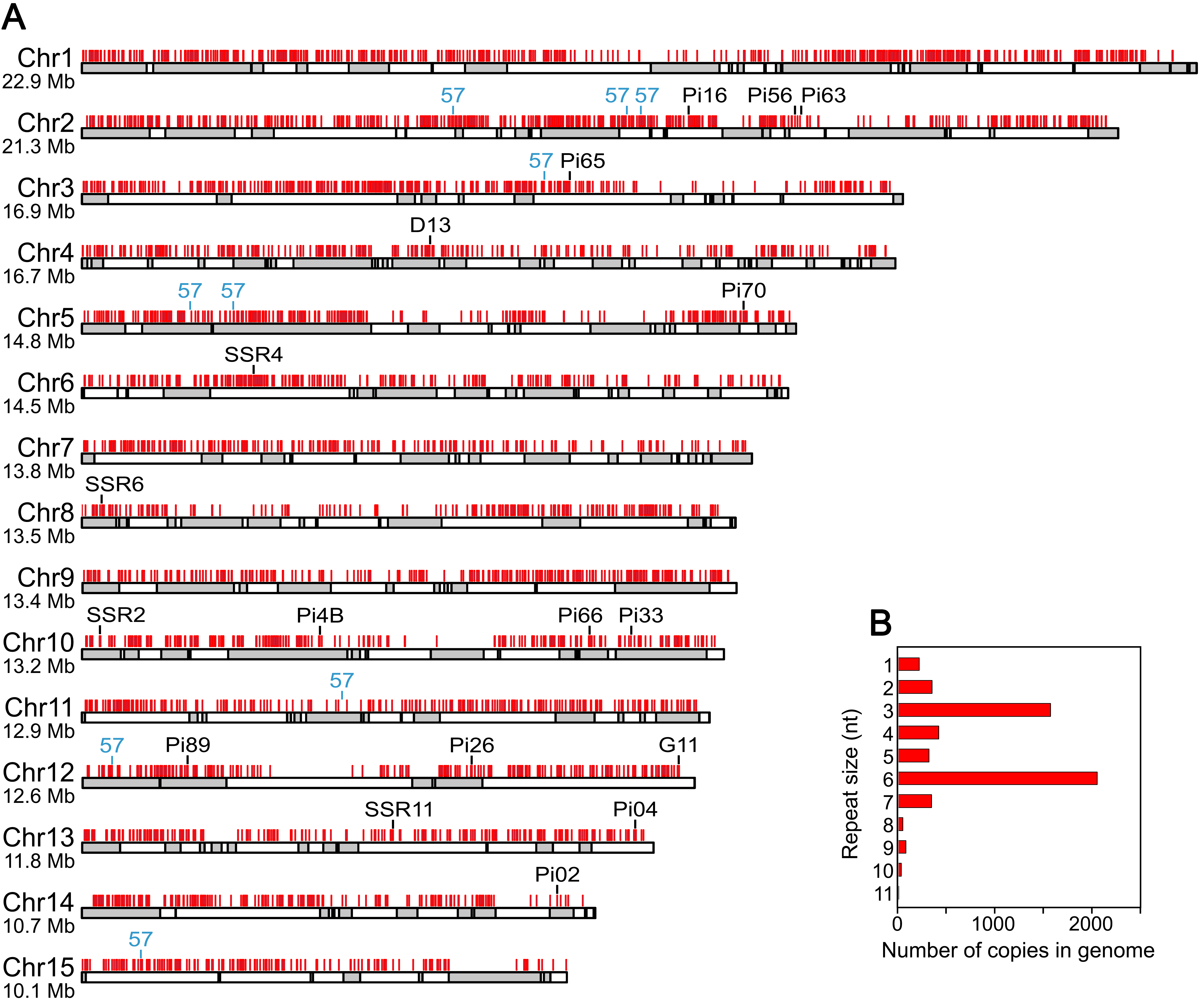

Supplement: S7 Fig — A, Total SSRs in the genome are indicated by red lines. SSRs unplaced on chromosomes, which represent 9.2% of the total, are not shown. As in Fig 2, the alternating white and grey blocks on each chromosome represent contigs from the Stitch7 assembly. Marked above the chromosomes are the locations of SSR markers used commonly in population genetics studies (black text) and RG57 ("57" in blue text). The SSR locations are based on sequences described by Lees et al. [23] and Li et al [122]. The RG57 locations were detected by searching the genome with GenBank accession JN160727. B, distribution of repeat unit sizes in SSRs. (TIF) [file ppat.1010869.s013.tif]

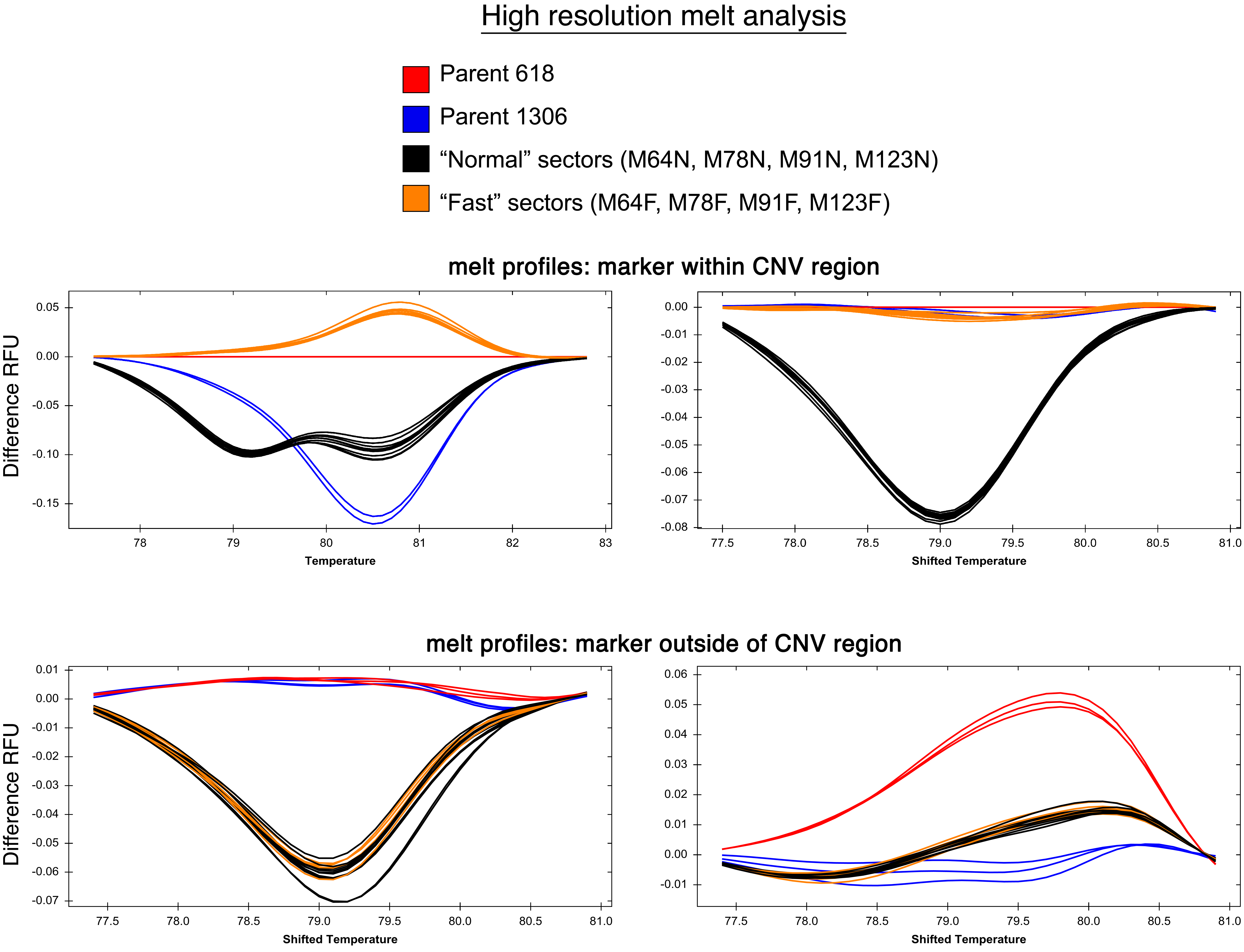

Supplement: S9 Fig — Melt profiles were developed for amplicons within and outside the region of Chr3 proposed to be deleted in the fast-growing sectors. The amplicons were generated using primers flanking sites that were polymorphic between the parents but homozygous within each parent. Thus, melt analysis should distinguish the homozygous (including monoallellic) and heterozygous (biallelic) states. The top two graphs show that melt profiles of an amplicon from the right end of Chr3 from normal and fast-growing sectors are distinct, with the fast-growing sector matching the homozygous parent. The same result was obtained for each pair of fast and slow-growing sectors from the four F1 strains. The lower two graphs, obtained for an amplicon from an undeleted region on Chr3, are consistent with its heterozygosity in both sectors. (TIF) [file ppat.1010869.s015.tif]
