# Supplementary material for: Karyotype variation, spontaneous genome rearrangements affecting chemical insensitivity, and expression level polymorphisms in the plant pathogen Phytophthora infestans revealed using its first chromosome-scale assembly
Source: PLoS Pathog. 2022 Oct 10;18(10):e1010869. doi: 10.1371/journal.ppat.1010869 (PMC9584435; doi:10.1371/journal.ppat.1010869)
Supplement: Attachment — Submitted filename: Review PPATHOGENS-D-22-01160.docx [file ppat.1010869.s016.docx]

The manuscript Karyotype variation, spontaneous genome rearrangements affecting chemical resistance, and expression level polymorphisms in the phytopathogen Phytophthora infestans revealed using its first chromosome-scale assembly by Matson et al., provide a detailed genetic-genomic analysis of Phytophthora infestans chromosomes. Despite the fact that it is high quality, detailed investigation at this point I don’t think it should be accepted for publication in PLoS Pathogens. As is the manuscript is definitely worth publishing in other venues and will be definitely be beneficial for the Oomycete community.

Using better computational analysis of previous long and short read sequencing combining with analysis of meiotic segregants and mapping of RNA sequencing the authors were able to form a better genome of P. infestans bringing it almost to chromosome level assembly. Using their analysis they were able to determine the ploidy of parental and segregant strains. The were able also to map genes and pathogenicity genes and found they are not distributed equally across the genome. The authors were also able to identify a segment in chromosome 3 that when loss in one of the homologs confers resistance to Metalaxyl.

My main concern is the descriptive nature of the manuscript and the fact that most of the data presented does not explain important observations regarding pathogenicity. Many of the major findings (ploidy, aneuploidy, gene density (two speed genome)) were previously reported as indicated by the authors themselves yet this manuscript does provide a more detailed description. The major contribution is the genetic/genomic analysis of Metalaxyl tolerance. To be accepted a much revised version of this manuscript should increase the resolution of Metalaxyl resistance showing exactly how the LOH in the regions contributes to the phenomenon (when such information is available I would suggest the authors to break the manuscript into two)

Minor concern is the genomic status of the meiotic progeny. While not an expert in P. infestans I thought it is mostly clonal and genetic recombination is rare in most of the world. I wonder is the parents used for the crossing are really the best model to study genetic crosses in this pathogen? Will the results of aneuploidy and ploidy would be different if other isolates were used?
