# Supplementary material for: Karyotype variation, spontaneous genome rearrangements affecting chemical insensitivity, and expression level polymorphisms in the plant pathogen Phytophthora infestans revealed using its first chromosome-scale assembly
Source: PLoS Pathog. 2022 Oct 10;18(10):e1010869. doi: 10.1371/journal.ppat.1010869 (PMC9584435; doi:10.1371/journal.ppat.1010869)
Supplement: Attachment — Submitted filename: PLOS_response to reviews.docx [file ppat.1010869.s017.docx]

**Responses to Editor and Reviewer comments**

(line number refer to the revised "clean" copy of the manuscript)

**Editor comments**

Reviewer 2 and 4 mention that the used parental strains and their offspring might not be typical for natural P. infestans populations. As proposed by reviewer 4, I would suggest to acknowledge the nature of the strains and to discuss the implications in the revised manuscript in more detail. Reviewer 4 also suggests to include a discussion, and potential comparison, of the new assembly to the telomere-to-telomere assembly of P. effuse (Fletcher et al. 2022).

As suggested, we provide more details of the strains (isolation year and host). We also discuss the potential utility of comparing our assembly with the more complete assembly of *P. effusa.*

Reviewer 1 suggests an additional computational analysis of the tRNA to study their evolution in oomycetes, which is an easy analyses worth to include.

As suggested, we have included a section that compares tRNAs in different oomycetes. The results are included in the text and a new supplemental figure. Please note that to avoid assembly artifacts, this was limited to the species that have assemblies in NCBI Genome based on long reads (PacBio or Nanopore).

Reviewer 2 and 4 both mention that additional analyses that would link ploidy changes to virulence would be particularly impactful - if already available, this data could indeed be included. I also agree with reviewer 4 that generating novel data to address this is beyond the scope of the manuscript, and thus I leave this decision to the authors' discretion.
We only have data on tuber infection. We intentionally decided to focus on tuber infections to focus on how ploidy would affect the ability to grow through the plant and defeat host defenses, while avoiding other complications in the phenotyping such as including the ability of zoospores to encyst, form appressoria, and penetrate the host cuticle. We acknowledge that the infection cycle on leaves involves an additional step (i.e. appressorium formation), and have added a statement (li 440) that whether these steps are affected by ploidy remains to be determined. Thank you for not requiring us to re-phenotype all of the progeny on leaves.

Reviewer #1: . . . . . I have no major comments, just several suggestions where minor improvements could be made to the manuscript . . . . . I think that readers would find it useful if the authors measured variation within and between tRNA families given their unusual expansion patterns.

As suggested, we have expanded our analysis of tRNAs. This has resulted in two new full-page multi-panel supplementary figures. Combined with additions to the text, we now illustrate both how different tRNA gene families have evolved within *P. infestans* and between different oomycetes.

. . . . and if the authors clarified exactly how they defined a “gene cluster”.

We defined a "cluster" as cases where genes were within 2.5 kb of each other. This was mentioned previously in the manuscript, but we have now added an additional sentence on that point in Methods. The definition is now provided on lines 215, 901, and 1424.

Reviewer #2: (No points to address)

Reviewer #3: (No points to address)

Reviewer #4: . . . There are a couple of areas that I felt could be explored:

1. A Peronospora full-length T2T assembly was published on BioRxiv in 2021 with indications of strong synteny and genomic organisation to Phytophthora genomes (Fletcher et al 2022). Although a comparison of the assemblies is not required at this stage of the current study, I feel the work of Fletcher et al should be acknowledged and the value of downy mildew versus Phytophthora comparisons flagged as valuable for future studies. This could, for example, be built into the discussion around L671-8.
As suggested, we have now included a discussion of the utility of performing comparisons with the *Pe. effusa* assembly (li 744). We appreciate the reviewer's comments that such a comparison "is not required at this stage of the current study". Our assembly is still somewhat fragmented and a comparison at this stage would probably lead to false conclusions about the conservation of chromosome structure and gene order.

2. Much of the discussion presumes that the behaviour of the laboratory strains and their progeny is typical of a population of diploid strains in nature. How can authors be sure that this is the case? . . .

The reviewer raises an interesting point. Therefore we have added in Discussion the statement "It would be interesting to learn if the frequency of ploidy variation observed in our laboratory studies would resemble that from a sexual population formed naturally within a potato field, or with strains from other regions of the world." (li 675)

We do believe that the phenomena described in our paper are real and not limited to our strains. Indeed, there are reports from other groups that changes in ploidy can occur during crosses (the Carter et al reference). How the frequency of such phenomena in nature compared to in our laboratory studies is unknown, and not all strains may behave the same as there is substantial genetic (and ploidy) diversity throughout the world. Since we decided to use three of the parents from the center of origin and genetic diversity of the species (Mexico), which are genetically diverse based on SSR analysis, we believe that our results are less biased than if strains from another region were used.

Caution needed as these strains have been maintained on agar under in vitro conditions for decades. It could be argued that this either represents stressed conditions (away from natural habitat) or ‘relaxed conditions’ with no plant infection or environmental stressors.

While the strains were collected decades ago (three by the Fry lab at Cornell), they have been in cryostorage for most of those years. We can not however exclude the possibility that mutations occurred while being cultured. There could also be bias associated with the original isolation of the strains, as some genotypes may be more easily recovered from infected leaves. This issue is addressed in the revised manuscript by our statement described above, "It would be interesting . . . " (li 675).

It would have been nice to include studies on that could advance our understanding of the biology and genetics of mating type amongst the progeny but perhaps that is planned for a future study?
L372-387 shows that the tested phenotypes of F1 isolates were remarkably robust to changes in karyotype. It would have been interesting to test more traits such as virulence but again that was beyond the scope of an already comprehensive paper.

We did attempt to identify SNPs linked to mating type, but unfortunately none were identified. There could be many explanations for this, such as the possibility that strains were mis-phenotyped, that there was a change in karyotype between the time when DNA was isolated and when the phenotype was determined, or that the inclusion of polyploids in the analysis confounded the linkage analysis.

Will the genome assembly be available somewhere that can be accessed by a Genome Data Viewer for the community to exploit?

The assembly is being uploaded now to NCBI under Bioproject Accession PRJNA868814. We will also make the 1306 assembly and annotation available to fungidb.org.

It would have been nice to have compared strain 1306 to T30-4 but I realise that this was probably beyond the remit of this paper.

We did make some initial comparisons between 1306 and T30-4, but decided that this would be nonproductive. Many of the major differences that we found were attributable to errors or gaps in the T30-4 assembly, and not necessarily to the strains themselves. We would like to avoid comparing genomes generated with different sequencing technologies or assembly programs.

Part III – Minor Issues: Editorial and Data Presentation Modifications

Reviewer #1: 57: I’m not convinced that this a truly “novel” observation. The authors themselves cite a previous study detailing this observation (lines 79-80)

True, this has been described before. We have therefore deleted "novel".

112: please specify what type of variation you are referring to here

Our study addresses so many areas of variation (SNPs, ELPs, ploidy, virulence, transpositions, etc) that we decided it would be simpler to delete "variation" from this sentence.

149: where did the genetic data for the 3 alternate parental strains come from? I see in the methods that they were sequenced using Illumina short reads and mapped to the reference, perhaps a brief explanation of that procedure is warranted here

As suggested, we added to the sentence " based on mapping Illumina reads to the Stitch7 assembly." (li 148) We also provided more details in Methods about the sequencing.

152: where did the data for these 82 progeny come from?

We sequenced these (plus the 32 from the other cross) as part of this project. This was previously described in Methods, but to make this clearer we reformatted that section to describe the Illumina sequencing of parents and progeny in a separate paragraph. (li 829)

209-210: the syntax of this sentence is non standard: it should read “549 and 128 genes were predicted to encode .. etc”

Thank you for the suggestion. We have rewritten this sentence but in a different way to avoid starting with a number.

213: the term “gene cluster” is more commonly used to describe metabolic gene clusters where genes encode different reactions in the same metabolic pathway. To avoid confusion, please describe these genes using a different term or with additional descriptors, perhaps “densely arranged clusters of genes”. Also, it would be useful to provide a definition of how these clusters of genes were defined both at its first mention and in the methods.

Thank you for pointing this out. Indeed, our use of the word "cluster" might be confusing to those working on non-oomycetes particularly fungi. Consequently, we have provided more details in Results and Methods to describe what we mean by a cluster: "a cluster is defined as genes spaced within 2.5 kb of each other, which may or may not have related functions or act in the same pathway." (li 215). Similar information is provided on lines 901 and 1424.

Figure 1: should the X axis be labeled ‘alternate allele’ and not ‘alternate read’, as it is in some later figures?

To be consistent, we have changed this to "Alternate read ratio" in Figs 5 and 10.

220-221: what data support this assertion?
We have modified the sentence to give the details: " which we presume contains a centromere based on its low gene density and the presence of the CoLT sequence." (li 222)

244-250: How genetically variable are different copies from the same tRNA family, and are there some families that are more variable than others?

Some families show very little variation, others show much variation. Coverage of this topic has been added to Results, and described in a new supplemental figure (Fig. S4). There are now three full-page figures on tRNAs in the paper (S2, S3, S4) which is probably as much as one would dare put in a Plos Pathogens paper.

378-381: The authors observe that diploids grow faster than any other karyotype. However, there appear to be pretty large differences in sample sizes between the different karyotype categories (e.g., there are ~40 diploids but only two 4N isolates). How might differences in sample sizes have impacted your ability to detect differences between groups? And can you please indicate the sample size of each Karyotype category in Figure 6?

The reviewer is correct that the small numbers of 3N+, 4N-, and 4 progeny, combined with the wide range of phenotypes in some of the classes, make it hard to detect small significant differences between some of the ploidy types. A comment to this effect has been added to the text: "The sensitivity of our analysis was however constrained by the small number of progeny containing nuclear contents above 3N (li 437). Also, as requested, we have added the sample sizes to Fig. 6.

392: please include some details about how many strains were examined for RNA expression exactly.

We examined both parents and 32 progeny. The number of progeny (32) was added to the text (li 451).

467-468: did these events actually result in changes to transcript abundance? It would be useful to report the fold changes of these examples specifically.

Zero transcripts were detected for these genes. Perhaps our use of the word "quiescence" was confusing. We have described the phenotype more explicitly: "Three examples which lacked detectable transcripts in the coding sequences of genes in strain 1306. (li 528)"

480: I’m a little confused about which ‘silent’ genes you are referring to here: perhaps some context would be helpful.

As suggested, we have modified the sentence to avoid confusion: "When all of the silent genes in 1306 and 618 were examined." (li 541)

Also, to which ‘family’ are you referring?

This referred to all of the "silent" genes in 1306 and 618. We have modified the sentence to include the word "overall" and we also added that the families were defined using OrthoMCL. (li 542)

681: do you mean the ease at which these mutations arose, or do you really mean to refer to the ease at which they were detected?

Yes, arose is the better term. We have made the change as suggested.

902-903: can you please provide some brief details about how this script works exactly?
A brief description of the method is provided in Methods, along with a literature citation (li 983 and reference 119).

Reviewer #2: (No Response)

Reviewer #3: I suggest that the authors change ‘chemical resistance’ to ‘chemical insensitivity’ in the title. This will be more in line with terminology in the rest of the manuscript.

We have made the suggested change.

Line 341 reports that Fig. 5C shows F1 strain 20.23, an aneuploid diploid (2N1-). In a closer examination of this figure panel, it looks as if it should be 2N3- (chromosomes 7, 8, and 15). Can the authors please check this.

Thank you for pointing this out. We have made the change.

Line 397. The authors state that it is ‘more common for mRNA levels to be higher in polyploids’. The normalized data (not just the fold-change) supporting this statement need to be shown in a supplementary table. The same comment applies to RNAseq comparisons between strain 618 and 1306.

The normalized data have been added to the supplementary tables. Perhaps the reviewer is also thinking that we are claiming that the amount of mRNA overall per nucleus is higher in polyploids; we are not making that claim. To make this more clear we reworded the sentence to say "more common for genes to have higher then lower mRNA levels in polyploids." (li 456)

Line 655. The authors speculate that there may be a selective advantage to nuclei undergoing ploidy reduction in culture or within a lesion. However, this is not borne out in reality, as the authors note in Introduction lines 96-97 that the majority of field isolates are triploid. It may be best to remove this speculative statement on lines 654-655.

We have reworded this sentence to more accurately expression our point. We meant that there were forces that might cause or nuclei with reduced nuclear content to appear within hyphae. This is totally separate from the forces that might give triploids a selective advantange in a potato field.

Line 695-696. Some more detail on the isolates used in this study is needed. What host plants were they originally isolated from, what year, and what culture collection(s) are they held in?

As suggested, we have added the additional details in Methods. (li 766)

Line 708. It is surprising that the disease score assays were only carried out on potato tuber slices. Late blight is primarily a foliar disease, so why was leaf infection not used? Sliced tuber tissue is effectively wounded tissue, so removes the early infection events from the analysis of pathogenicity. Tuber slice assays will only reveal the extent to which hyphae can colonize tissue. This should be pointed out in the Results.

As suggested, we have added to Results the sentence: "It remains to be determined whether other stages in the disease cycle are affected by ploidy, such as appressorium development which occurs on leaves and not tubers." (li 439)

Line 754. Literature citation for the ‘Chicago method’.

Added.

Line 755. Geographical location for Dovetail.

Added.

Line 756. Geographical location for Cofactor Genomics.

Added.

Line 763. Insert ‘.’ into Chimericognizer [90] The data

Done.

Line 765. Literature citation for BUSCO.

Added.

Line783. Markers that showed

We have made the suggested change

Line 784. Literature citation for genotype-corrector.

Added.

Line 858-859. Which Illumina sequencer was used? HiSeq, NextSeq, MiSeq?

We have inserted HiSeq in Methods.

Line 880-881. More detail is needed for the RNA extraction method than just ‘using phenol/chloroform’. Please describe the method or give a literature citation for it. The same comment applies for the polyacrylamide-urea gel electrophoresis; please describe the conditions or provide a literature citation.

We have provided more details as requested.

Line 912. Manufacturer needed for DNase.

Added.

Line 914. Is the full gene ID PITG_09862? What is the function of the protein encoded by this gene? Can a GenBank accession be given for it?

This gene encodes a Kelch repeat protein. We have provided the accession number and the literature reference that described its identification as a useful control. (li 1000)

The formatting of references should be checked. Some organism names need to be in italic font.

We have (hopefully) made the necessary corrections. Some gene names also needed to be italicized.

The figures in the manuscript are in some instances necessarily complex. As a general comment there needs to be more detail given in the figure legends to help the reader interpret them without having to delve into the article text to find the information.

We have modified many of the legends to provide additional information, including those listed below:

Figure 1. What does ‘density’ on the Y axis describe?

This is a kernel density estimation plot. This is essentially a fundamental data smoothing approach where inferences about a population are made, based on a finite data sample.

We changed the legend to specifically indicate that this is "Kernel density", as well as to add other information. (li 1345)

What is the red trace line in each graph? What does the pink shaded area represent?

We have added this information: red is kernel density, pink is a normalized distribution.

Figure 2F. What do the orange or red horizontal lines in some of the chromosome bars represent?

As panel F only has grey bars, we suspect that the reviewer is referring to the partially overlapping triangles in panel A. In the low resolution image provided to the reviewers, these might appear to be bars. However, in the higher resolution image that will be in the published version, these can be discerned as representing individual but closely-spaced triangles.

Figure 3. The legend for this figure is particularly minimal. There needs to be more detail about what is shown in the layout for each chromosome. State the significance of the ‘forked’ mapping seen for some linkage groups.

We apologize for this poorly described legend. We have added many more details. Also, the significance of the forked bars (evidence of chromosomal rearrangements) was mentioned in Results in the original manuscript (li 179).

Figure 5D legend. Line 1285. Correct (Chr3, Chr4, Chr7, Chr9).

Corrected.

Line 1287 states that this is for Chr3, but Chr9 is shown in the figure.

Corrected.

Reviewer #4: There are a few minor points to address as listed below.
Are all gene names in this study the same as those from other work with PITG gene names? I see this in mentioned in L867 but it is not explicit. Could this be clarified and if not the case, then a look-up table needs to be included to allow comparisons.

Yes, they are the same and we apologize for the confusion. To increase clarity, at the first place where gene numbers are provided we added "(please note that this paper refers to genes by their corresponding numbers in the Broad Institute database, without the "PITG" prefix) (li 524). This is also stated in Methods.

Fig S5 and text L292-300. This figure is a great idea but the key SSR markers in the 12-plex set of Li et al (2013) including some from Lees et al (D13, Pi4B and Pi63) are missing. It would be helpful to also include the SSRs below. The claim in L298 that “while eight chromosomes lacked any marker” may be untrue if these additional 8 markers are added.

Thank your for the suggestion; we had only included SSR markers from one paper. We have added in the additional markers, and the Li et al reference. SSR8 did not have any perfect matches in our assembly (or the T30-4 assembly) and thus its position is ambiguous; this is stated in Results.

Similarly, with L630-1 “only sample selected chromosomes”.

This has been this modified to say that 5 chromosomes lack an SSR marker and several of the markers are tightly clustered (li 352).

Lastly, an explanation of the letters following the RG57 marks in Fig S5 would be helpful and please state what the grey bars on the chromosomes represent.

The letters were just different hits in the genome; they have been deleted. The white and grey sections represent separate contigs from the Stitch7 assembly, as stated in the Fig. 2 legend, but also now said in the new Fig S5 (now S7) legend.

L456 “no polymorphisms were sometimes detected” cannot detect something that does not exist - suggest “a lack of polymorphism in some case which may implicate…”

We have corrected the grammar: " polymorphisms were sometimes not detected " (li 516)

L695-6 year of isolation of the strains should be added.

Added.

L945 dna vs DNA

Corrected

L1122 and L1000 italics missing

Corrected

**Reviewer 2** (referring to comments in the attachment)

To be accepted a much revised version of this manuscript should increase the resolution of Metalaxyl resistance showing exactly how the LOH in the regions contributes to the phenomenon (when such information is available I would suggest the authors to break the manuscript into two)

The reviewer raises the intriguing approach of obtaining many more Chr3 deletion strains. This is an excellent idea, as it would allow finer-scale mapping of the resistance locus. We believe that such work is beyond the scope of the current manuscript, especially since as the reviewer notes this would likely require splitting the manuscript into two (or three considering other additions made in response to suggestions of the other reviewers).

Minor concern is the genomic status of the meiotic progeny. While not an expert in P. infestans I thought it is mostly clonal and genetic recombination is rare in most of the world. I wonder is the parents used for the crossing are really the best model to study genetic crosses in this pathogen? Will the results of aneuploidy and ploidy would be different if other isolates were used?

These are good points. Three of the strains (550, 618, and 6629) were genetically diverse isolates obtained from regions of Mexico in which sexual populations are found. Also, and perhaps more significantly, Mexico is believed to be the center of origin of *P. infestans.* Since we observed aberration segregation involving chromosomes from all four parents, three of which came from the center of origin of the species, we propose that the phenomenon is widespread. However, another region in which sexual recombination is common is Scandinavia, and it is possible that aneuploid or polyploid progeny arise at a different frequency there. Thus, we added the sentence "It would be interesting to learn if the frequency of ploidy variation observed in our laboratory studies would resemble that from a sexual population formed naturally within a potato field, or with strains isolated from other regions of the world." (Li 676).
